# Chlorophyll Fluorescence in Wheat Breeding for Heat and Drought Tolerance

**DOI:** 10.3390/plants13192778

**Published:** 2024-10-03

**Authors:** Firuz Abdullaev, Polina Pirogova, Vladimir Vodeneev, Oksana Sherstneva

**Affiliations:** Department of Biophysics, National Research Lobachevsky State University of Nizhny Novgorod, 23 Gagarin Avenue, 603022 Nizhny Novgorod, Russia

**Keywords:** breeding, chlorophyll fluorescence, PAM fluorometry, crop yield, stress tolerance, drought, heat, wheat

## Abstract

The constantly growing need to increase the production of agricultural products in changing climatic conditions makes it necessary to accelerate the development of new cultivars that meet the modern demands of agronomists. Currently, the breeding process includes the stages of genotyping and phenotyping to optimize the selection of promising genotypes. One of the most popular phenotypic methods is the pulse-amplitude modulated (PAM) fluorometry, due to its non-invasiveness and high information content. In this review, we focused on the opportunities of using chlorophyll fluorescence (ChlF) parameters recorded using PAM fluorometry to assess the state of plants in drought and heat stress conditions and predict the economically significant traits of wheat, as one of the most important agricultural crops, and also analyzed the relationship between the ChlF parameters and genetic markers.

## 1. Introduction

Wheat is one of the most widely cultivated crops. It is critical to human nutrition, accounting for about 18% of all calories and up to 19% of protein consumed [1]. Simultaneously, with the growing population and the increasing need to step up the pace of production, there is a reduction in arable land, which further increases the requirements for crop productivity. Not all regions in which wheat is grown have favorable climatic conditions, and therefore grain yield (GY) is largely limited by abiotic stressors such as drought and elevated temperatures [2]. In response to demands from agricultural producers, breeders are working to develop new wheat cultivars that can provide high yields, including when grown in unfavorable conditions [3,4].

Currently, traditional approaches to the selection of genotypes for the development of new crop cultivars are enhanced by the use of genomic and phenomic technologies [5,6,7]. The latter are currently regarded as an essential element of breeding programs. At the same time, protocols for introducing phenotypic methods into the system for selecting promising genotypes are still being developed and improved. In particular, optical phenotyping methods are actively being introduced into this process [8,9]. Optical methods include spectral methods, in particular, multi- and hyperspectral techniques, thermal imaging, the assessment of photosynthetic activity based on chlorophyll fluorescence (ChlF) parameters, and others. Methods based on the recording and interpretation of data on the intensity of ChlF have long been firmly established in the practice of plant research [10,11,12,13]. These methods include the pulse-amplitude modulated (PAM) fluorometry, the fast polyphasic rise of the induction curve (OJIP), fast repetition rate (FRR) fluorometry, etc. [14,15,16]. Their use in the breeding process is a promising approach to assess the health of plants at early stages of development and select genotypes that have the greatest potential to achieve target traits.

One of the most informative methods for recording ChlF is the PAM fluorometry. The PAM parameters, calculated based on the ChlF intensity, reflect the activity of numerous processes occurring in the photosynthetic apparatus [10,11,12]. It is known that the PAM parameters are closely related to CO₂ assimilation [15,17]. An analysis of the changes in ChlF parameters makes it possible to detect changes in the physiological state of plants in response to the influence of various factors, including the availability of water and minerals, temperature, lighting, biotic factors, etc. [18,19,20,21,22,23,24].

This review discusses the potential and examples of the use of PAM fluorometry in the breeding process, both for predicting significant wheat traits, including in combination with genotypic predictors, and for assessing the current plant state in various conditions, including field studies and stress modeling in a controlled environment. Due to the larger amount of data available, we analyze the results obtained on *Triticum aestivum* (unless otherwise noted).

## 2. Briefly about PAM Fluorometry

PAM fluorometry is based on recording the intensity of ChlF under different light conditions [25]. In this case, such parameters as the minimum level of fluorescence under weak measuring light and the maximum level of fluorescence during a saturation pulse of high intensity light in a dark-adapted state (F_0_ and F_m_) and under the actinic light (F_0′_ and F_m′_), as well as the current fluorescence level under actinic light (F_t_), are recorded. In the simplest case, F_0_ is recorded after a period of dark adaptation; then, a pulse of saturation light is applied, causing the closure of all working photosystems (electron acceptors Q_A_), and F_m_ is recorded [26]. F_v_/F_m_ (the maximum quantum efficiency of photosystem II (PSII)), calculated using F_m_ and F_0_ values, is the most commonly used PAM parameter for assessing the condition of plants [22,27,28,29,30]. The PAM method is also used to study the processes accompanying changes in fluorescence intensity after the actinic light is turned on (Kautsky effect), which reflects transient processes, including the rate of activation of the Calvin–Benson cycle, as well as photosynthetic activity in the light-adapted state [31,32,33,34]. Using the PAM method, it is possible to evaluate the contribution of the photochemical and non-photochemical quenching of fluorescence to the realization of the energy of absorbed light [32,34]. The most commonly analyzed PAM parameters are shown in Table 1.

Among the advantages of PAM fluorometry, it is important to note the high information content and sensitivity of PAM parameters, which allows for a comprehensive assessment of the state of the photosynthetic apparatus and makes the PAM method one of the most used for studying the activity of photosynthesis. On the other hand, it has some limitations. Systems using PAM technology have a relatively high cost due to the technical complexity of the integrated equipment, but manufacturers have devoted much effort to making PAM fluorometers more affordable and easier to operate [36]. A major limitation is the relative low speed and, in some cases, the methodological complexity of obtaining data due to the fairly long period needed (tens of minutes) for dark adaptation [37]. This factor does not allow for the use of PAM fluorometry in studies comparable in throughput to other optical methods, such as the registration of solar-induced fluorescence or reflectance parameters. In addition, researchers encounter difficulties in interpreting PAM parameters. The complex structure of the crown and individual leaves in vascular plants, as well as a number of other features, make it difficult to interpret the results obtained [13]. At the same time, such features themselves are of great interest for study. Attempts to reduce the impact of these limitations on the use of this method are currently being made. PAM-imaging systems, making it possible to record ChlF parameters simultaneously on a lot of plants or on a wide area of a large plant, have become widespread. In addition, attempts to record initial photosynthetic parameters to estimate F_v_/F_m_ without prior dark adaptation using machine-learning methods are being made [38]. The development of technologies and approaches to recording and interpreting ChlF data enhances the prospects for using PAM fluorometry for large-scale plant studies. Combined with this fact, extended information about the state of plants and their response to external factors [11,12,39] makes methods based on recording ChlF, among which PAM fluorometry is the most widely used, promising for use in the breeding process. PAM parameters can be used to assess the sensitivity of various genotypes to stressors, acting both as independent predictors of stress tolerance and in combination with other phenotypic and genotypic markers.

## 3. Detection of Drought and Heat Stress Using ChlF Parameters

PAM fluorometry, which makes it possible to assess the current level of photosynthetic activity, is widely used to assess the effects of stress. This is due to the fact that photosynthesis is a complex process that is highly sensitive to environmental conditions [40]. Moreover, the rates of photosynthesis directly affect plant productivity, so the maintenance of its activity serves as an indicator of potential survival and yield under stressful conditions [41]. Changes in photosynthetic activity can be caused both by the direct influence of environmental factors on the functioning of the photosynthetic apparatus, and by changes in other physiological processes that are closely related to photosynthesis, including transpiration, the transport of assimilates, etc. [42]. Thus, the use of methods based on recording ChlF allows researchers to obtain fairly complete and accurate information about the state of the photosynthetic apparatus under the influence of stressors of various natures [12,23].

### 3.1. Drought Stress

Drought is one of the main causes of crop losses in agricultural plants, including wheat [43,44,45]. At the same time, researchers face the challenge of studying the state of plants under conditions of water deficiency before the appearance of irreversible changes caused by drought [46]. Due to the sensitivity of the light-dependent reactions of photosynthesis to changes in water content, ChlF parameters are widely used to assess plant responses to drought. At the same time, various ChlF parameters can act as criteria for assessing the susceptibility of plants to drought-induced changes [12].

F_v_/F_m_ is one of the traditional ChlF parameters used to diagnose the state of plants. As a rule, under optimal conditions, the value of F_v_/F_m_, which reflects the maximum quantum yield of PSII, ranges from 0.77 to 0.84 in different plants [47,48,49,50]. The convenience of measuring this parameter due to its independence from the current light intensity and gas composition of the air determines its frequent use to assess the effects of various stressors. F_v_/F_m_ decreases under water deficiency, but sensitivity to the intensity of water stress varies among environments. Thus, a 15-day soil moisture deficit in 6-week-old wheat plants grown in greenhouse conditions caused a decrease in the F_v_/F_m_ level from 0.73 to 0.49 [51].

At the same time, the sensitivity of the parameter F_v_/F_m_ to drought depends on the strength and duration of water deficit. In particular, no differences were found between F_v_/F_m_ in rainfed and irrigated conditions in the field [52]. F_v_/F_m_ also did not change significantly in response to soil drought stress in pot experiments [53,54]. In addition, F_v_/F_m_ responds poorly to moderate drought or early stages of severe drought stress [53,54,55]. Moderate physiological drought caused by polyethylene glycol did not cause changes in F_v_/F_m_ at medium N supply, while more severe drought significantly reduced the value of this parameter [56]. Due to the high stability of F_v_/F_m_, its use as a criterion of genotype tolerance at early stages or under moderate water stress seems unpromising. A decrease in F_v_/F_m_ in many cases is associated with irreversible damage to the photosynthetic apparatus, including the inactivation of photosynthetic reaction centers [57], while the activity of photosynthetic processes changes long before the onset of structural rearrangements in photosystems [58]. At the same time, the simplicity and high reproducibility of measuring F_v_/F_m_ both in laboratory and field conditions make it possible to propose it as a quantitative criterion for the depth of stress that a specific genotype can withstand during selection screening. Thus, this parameter is convenient for determining critical threshold values of drought tolerance of a genotype.

ChlF parameters, which reflect not the integrity, but the efficiency of the photosynthetic apparatus, are more sensitive to the effects of various stressors, including drought. These parameters primarily include Φ_PSII_, qP, ETR, as well as qN and NPQ [51,56,59]. Significant changes in these parameters occur even with a relatively weak change in water status.

Φ_PSII_ is one of the main ChlF parameters characterizing the efficiency of using the energy of absorbed light into photochemical processes; the parameter qP, which is close to Φ_PSII_, reflects the proportion of open photosystems II capable of participating in electron transport along the photosynthetic electron transport chain (PETC) [15]. Water deficiency causes the suppression of the linear electron flow, which manifests as a decrease in the values of the Φ_PSII_ and qP parameters [51,60,61,62,63,64]. In particular, a 10-day drought caused a significant decrease in Φ_PSII_ in winter wheat plants at different stages of development; Moreover, the depth of stress changes increased with increasing drought intensity [60]. The dependence of qP on drought intensity has also been shown. In particular, a significant suppression of qP was observed starting from day 6 of drought stress and statistically significantly increased with increasing treatment duration [62]. Along with pot experiments, a decrease in Φ_PSII_ and qP in wheat has also been observed under rainfed conditions in the field [65], as well as when physiological drought is induced by PEG [56,66]. It is worth noting that these parameters show similar dynamics and comparable percentage changes during drought. However, their level depends on the intensity of light supplied to the plant [17]. To take into account the intensity of actinic light that acts when recording ChlF parameters, the parameter ETR, which characterizes the rate of electron transport along the PETC, is used [49]. In general, the direction of drought-induced changes in ETR coincides with those for Φ_PSII_ and qP. However, the sensitivity of these parameters may vary due to genotypic differences and recording conditions [51,52,56].

A group of ChlF parameters that reflect the thermal dissipation of energy from absorbed sunlight, such as NPQ and qN, also show significant changes during drought stress development. These parameters have fairly high variability and sensitivity to changes in external conditions both in controlled and field conditions in *T. aestivum* [51,53,56,60,62,65,67]. The parameters NPQ and qN show an increase even with a moderate water deficit. In particular, qN in plants subjected to water deficiency was higher compared to the control after 4 days of treatment in a pot experiment [53]. Moreover, the level of qN and NPQ increases significantly with increasing drought duration [60,62]. It should be noted that along with the typical increase in NPQ during drought, a decrease in this parameter can be observed at the terminal stage of drought [68,69].

Drought-induced changes in the described parameters in *T. aestivum* begin at different stages of water stress, which leads to their different sensitivity and applicability. In most cases, ChlF parameters reflecting the efficiency of linear electron flow in PETC respond to water deficiency earlier than others (for example, F_v_/F_m_) [69]. This is due to the high sensitivity of electron transport to fluctuations in the activity of physiological processes and the rapid response to changes in signaling cascades. An increase in the intensity and/or duration of drought stress causes rearrangements and damage to the structure of photosystems, which manifests itself as a decrease in F_v_/F_m_. The further development of a drought of extreme intensity can cause damage to thylakoid membranes, which leads to a drop in the pH gradient and a decrease in NPQ, along with a reduction in photosynthetic activity.

The observed changes in ChlF parameters under drought stress in higher plants, including wheat, may be a result of changes in both the activity of the photosynthetic apparatus and other physiological processes. The complex of mechanisms that generate a signal of the water deficiency and cause changes in stomatal aperture includes the involvement of a large number of signaling molecules, the key of which are abscisic acid, calcium ions and reactive oxygen species (ROS) [70]. As a result of drought-induced stomatal closure, the availability of CO_2_ is reduced, which, together with a decrease in mesophyll conductivity for CO_2_ and the activity of enzymes such as RuBisCO, has a negative effect on the activity of the Calvin–Benson cycle and suppresses the entire process of photosynthesis, including the light-dependent stages [71]. The inhibition of PETC leads to the excessive formation of ROS, which, on the one hand, perform a signaling function, triggering numerous cascades of reactions; on the other hand, ROS cause damage to thylakoid membranes and proteins of the photosynthetic apparatus, leading to a decrease in the parameters of photosynthetic activity [72]. Such changes may be irreversible and require de novo synthesis of a number of photosynthetic proteins.

Considering the high sensitivity of ChlF parameters to the action of drought stress, it is necessary to take into account that drought is a slowly developing stress factor under natural conditions. The strategy for developing drought tolerance includes, among other things, rearrangements in the photosynthetic apparatus [73,74]. This is also reflected in the ChlF parameters recorded during drought. On the other hand, the complexity of the parameters helps to identify features of drought tolerance mechanisms in specific genotypes [73], which can be useful in breeding.

### 3.2. Heat Stress

One of the most important impacts of climate change is the earlier onset of persistently high temperatures during the growing season [75]. Also, over the past century, the average annual temperature in regions where wheat and other major crops are grown has increased by 1 °C [76]. Wheat, like many C3 plants, has a temperature optimum for active photosynthesis, growth and development in the range from 17 to 25 ℃ [77]. At the same time, a significant part of wheat cultivation areas is located in regions where wheat is exposed to heat stress.

One of the most popular ChlF parameters used to assess heat stress in wheat is F_v_/F_m_. It makes it possible to assess the depth of stress in plants and the degree of damage to the photosynthetic apparatus. In particular, moderate heating for 5 days caused a decrease in F_v_/F_m_ in wheat plants; Moreover, the value of this parameter reached the control level after the cessation of the stressor [78]. The promise of F_v_/F_m_ for detecting stress changes during short-term exposure to a stressor was also shown in other works. In particular, F_v_/F_m_ acted as a sensitive indicator of the development of heat stress of varying intensity, as well as the recovery of photosynthetic capacity after its end [79]. In the work [80], a temperature increase by 2–3 °C for 4–8 days caused a decrease in F_v_/F_m_, and the magnitude of such a decrease varied in different years of the experiment. A long-term moderate increase in growing temperature can also negatively affect to the F_v_/F_m_ level [81]. Taking into account the fairly high sensitivity and convenience of recording F_v_/F_m_ both in laboratory and field studies, it should be noted that the F_v_/F_m_ level characterizes the functional integrity of the photosynthetic apparatus, which changes quite quickly when exposed to elevated temperatures. At the same time, its change is preceded by a change in ChlF parameters, which reflect the current activity of photosynthesis, including electron transport through the PETC. In particular, exposure to heating to 40 °C for 30 min did not cause changes in F_v_/F_m_, while a more sensitive parameter, NPQ, was significantly reduced in *T. aestivum* [82]. The physiological basis of F_v_/F_m_ tolerance to mild or early-stage heat stress may be due to changes in the functioning of PETC, which are aimed at protecting PSII from damage. It was shown that short-term heating in the dark (30–60 min) caused a decrease in the F_v_/F_m_ value, which was due to the degradation of the D1 protein [83]. At the same time, damage to PSII did not occur during similar heating in light, which may be due to the intensification of non-photochemical quenching and the activation of cyclic electron flow. Such changes lead to a decrease in the linear electron flow, which manifests as a drop in the parameters Φ_PSII_ and qP [84,85,86,87]. The activation of the mechanisms protecting the photosynthetic apparatus from damage during heating causes a later response of F_v_/F_m_ to the development of stress and, as a consequence, lower sensitivity to changes compared to parameters characterizing the activity of electron transport in PETC.

The parameters reflecting the intensity of the linear electron flow are very sensitive to changes in ambient temperature. In particular, a short-term gradual heating of wheat leaves caused the response of Φ_PSII_ in the physiological temperature range (when moving from 25 to 30 °C) during the first minute [88]. In this case, a moderate short-term rise in temperature caused an increase in Φ_PSII_, followed by a decrease with a further temperature growth (on average, when the temperature reached 40–45 °C) [88,89]. Heating wheat leaves to 40 °C for several hours caused a drop in Φ_PSII_ and ETR by about half [90]. Similarly, increasing the temperature in the phytotron to 40 °C for 4 h induced a decrease in Φ_PSII_ and qP [91].

The decrease in linear electron transport rate caused by moderate thermal exposure is usually accompanied by an increase in non-photochemical quenching [90,91,92]. At the same time, more intense exposure may form a different picture of the photosynthetic response. In particular, the short-term (30 min) heating of wheat grown hydroponically to 44 °C caused a slight increase in the energy-dependent component of NPQ (NPQ_F_), accompanied by a statistically significant decrease in Φ_PSII_; at the same time, exposure to a temperature of 46.5 °C caused a simultaneous decrease in both Φ_PSII_ and NPQ_F_ [86]. Similar results (a simultaneous decrease in both Φ_PSII_ and NPQ) were also shown in a pot experiment after heating wheat seedlings to 45 °C for 30 min [93]. Along with intense short-term heating, long-term exposure to lower (but still very elevated) temperatures (38–42 °C) can also induce such changes in the activity of the light-dependent stage of photosynthesis [94,95].

It is worth noting that variations in the tolerance of different genotypes of *T. aestivum* to elevated temperatures are also manifested in different stability of the photosynthetic apparatus. Such differences appear both in the short-term [29,87,88,93,96] and long-term [29,81,84,85] heat stress.

The main targets for the effect of heat stress on photosynthesis are the Calvin–Benson cycle, the oxygen-evolving complex, PETC and photophosphorylation [97]. In particular, when the temperature changes, the activity of RuBisCO, RuBisCO activase, and enzymes involved in the regeneration of ribulose-1,5-bisphosphate significantly changes [98,99]. This effect is caused by going beyond the temperature optimum, a decrease in ATP content, and oxidative stress. In addition to the reactions directly involved in CO_2_ assimilation, an increase in temperature can affect the functioning of the PETC; at the same time, PSII is more susceptible to heat damage compared to PSI. The main targets here are the D1 protein, the plastoquinone pool, cytochrome b559, the oxygen-evolving complex, etc. [100]. When exposed to critical temperatures, a decrease in chlorophyll content and the destruction of the lipid bilayer of membranes and protein structure can occur [101]. All of these processes lead to a decrease in photosynthetic activity, which is detected with a high sensitivity using ChlF parameters.

It is worth noting that plants have powerful adaptation mechanisms for survival in conditions of elevated temperatures, including the maintenance and recovery of photosynthetic capacity. The disclosure and regulation of such mechanisms will help optimize the heat tolerance of crop plants, particularly wheat, to improve agricultural efficiency [101].

In general, considering the efficiency of PAM fluorometry for assessing the depth of drought and heat stress on wheat plants, the following can be summarized: ChlF parameters are sensitive indicators for these stress factors. Their changes were observed at different stages of stressors of different intensity. The earliest changes are typical for parameters reflecting PETC efficiency (Φ_PSII_, qP, ETR) and non-photochemical quenching (NPQ, qN). It should be taken into account that Φ_PSII_, qP and ETR are characterized by a decrease with increasing stress depth, whereas NPQ and qN are characterized by a rise at the initial stages of stress development with a subsequent drop with increasing stress intensity and depth. F_v_/F_m_, reflecting the integrity of PSII, responds to the stressor later in comparison with the above parameters, but at the same time has a high reproducibility of measurements, which underpins its wide use. These features should be taken into account when developing specific protocols for assessing the state of plants under stress conditions.

## 4. Predicting Crop Traits Using ChlF Parameters

In addition to the detection of stress states in agricultural plants, the use of phenotypic traits recorded by non-invasive optical methods for the selection of potentially productive and stress-tolerant genotypes in the breeding process is in active development [102,103,104]. In particular, the work [93] showed that the quantum yield of PSII in the light-adapted state (Φ_PSIIef_) in young wheat seedlings was positively correlated with the accumulation of plant fresh weight at a later age. In addition, a parameter demonstrating the rate at which the quantum yield reached a steady-state value after turning on the light (half-time) was negatively correlated with the fresh and dry weight of older plants. The steady-state and transient parameters of photosynthetic activity reflect the rate of the biosynthesis process, which influence the final characteristics of mature plants. It is known that the accumulation of biomass in plants that have not reached maturity can be associated with the biomass of mature plants [105], and that, in turn, with the yield [4,106,107]. ChlF parameters also act as predictors of significant traits for other plant species [108,109,110]. Thus, the use of a combination of several ChlF parameters in conjunction with machine learning made it possible to predict the accumulation of fresh weight in lettuce plants [111]. In addition to the value of aboveground biomass, ChlF parameters show potential for predicting yield-related traits. In particular, F_0_ and F_v_/F_m_ were significantly correlated with wheat GY under well-watered conditions in some types of environments [52]. It is important to take into account that ChlF parameters recorded at different stages of plant development have different potential for prediction. In particular, such an effect has been clearly demonstrated in barley plants [112].

When assessing the potential tolerance of wheat to adverse environmental conditions, such as drought and elevated temperatures, researchers focus on two types of predictors based on ChlF. In the first case, phenotypic parameters recorded in plants under optimal conditions act as predictors [52,93]. In this case, the predictive potential of the parameters used is determined by the initial activity of the photosynthetic apparatus. In laboratory conditions, this effect was shown when assessing the potential tolerance of wheat seedlings to elevated temperatures, which acted as a short-term stressor, and to soil drought (longer-term stress) [93]. Tolerance to soil moisture deficit showed a strong negative correlation with the NPQ value in the light-adapted state and the maximum NPQ level after turning on the actinic light in younger non-stressed plants; the heat tolerance was positively correlated with F_v_/F_m_ and a dark level of Φ_PSII_ (5 min after turning the actinic light off). It is worth noting that the predictive potential of ChlF parameters recorded in non-stressed plants requires in-depth research. In particular, it may be limited by the fact that the entire spectrum of genotypic variations affecting the physiological state and final agronomic traits of plants under stress conditions may not be manifested.

The second type of tolerance predictors are ChlF parameters recorded in plants that have already been exposed to stress factors, for example, in uncontrolled field conditions. In this case, the relationship between the phenotypic parameters of young plants under adverse conditions and their final economically significant traits may be due primarily to the stability of the photosynthetic apparatus and plant defense systems, including the work of heat shock proteins, the antioxidant system, etc. In particular, under rainfed (water-limited) conditions, F_0_ and F_v_/F_m_ recorded at anthesis can show a statistically significant correlation with GY, but such a relationship can be ambiguous and unstable when growing plants in the different environments [52]. Also, the relationship of potential fluorescent predictors with yield and its components can be assessed by a set of phenotypic and agronomic characteristics recorded under conditions of different water availability. For example, in the work [113], the ChlF parameters F_0_′, F_m_^′^, Y (NO), NPQ, ETR_max_ and Y (II), recorded at the stage of grain filling, significantly correlated with GY, harvest index, the number of kernels per spike, and 1000 kernel weight in two water conditions (well-watered and water-limited conditions). At the same time, the strongest positive relationship with GY in wheat was shown for F_m_′ and Y (NO), negative—for NPQ.

In general, the use of phenotyping for the selection of promising genotypes at stages of plant development prior to full maturity in the breeding process opens up broad prospects for accelerating and optimizing the wheat breeding process. ChlF parameters are highly informative indicators of plant physiological state. In addition, the photosynthetic process and the accompanying changes in ChlF parameters are quite well studied, which facilitates the elucidation of the mechanisms of the relationship between identified predictors and target traits. At the same time, the use of several types of predictors is promising. Such predictors may include steady-state ChlF parameters, as well as parameters characterizing the light-induced dynamics of the efficiency of PETC operation, both under optimal and stressful conditions. In addition to a wide range of traits that can be studied at a fairly high speed using high-throughput phenotyping systems [114], the approach of integrating two interrelated components (phenotyping and genotyping) into the early selection process is being actively introduced [115,116,117]. This approach increases the reliability and accuracy of prediction, and therefore it is important to consider the available information on the relationship between ChlF parameters and genetic markers.

## 5. The Relationship between ChlF Parameters and Genotypic Characteristics

Bread wheat (*Triticum aestivum* L.) is an allohexaploid, which includes three genomes, A, B and D, with a size of 16–17 Gb [118]. The genome size of wheat is many times larger than that of other plant species [119]. Wheat, like other important agricultural crops (barley and rye), belongs to the Triticeae tribe. These crops have undergone many changes during the process of domestication [118,120]. The complete genome sequence of *T. aestivum* was formed as a result of hybridization of three diploid ancestors. The first hybridization event occurred between *T. urartu* (AA) and a yet unidentified species with a B genome, a close relative or ancestor of *Aegilops Speltoides*, with the formation of the tetraploid *T. dicoccoides*. The D genome was added to the domesticated tetraploid *T. dicoccoides* from *Ae. Tauschii*, forming the complete hexaploid genome of *T. aestivum* [118]. The D genome is characterized by fewer polymorphic markers, which indicates less genetic diversity due to a low recombination frequency [119,121]. The wheat D genome plays a key role in tolerance to biotic and abiotic stress, and also ensures the high baking qualities of wheat grain [122,123]. The three wheat genomes have approximately the same percentage of transposable elements (86%, 85% and 83% of the sequence for A, B, and D, respectively) [120]. However, the sizes of the genomes differ: B genome (5.18 Gb), A genome (4.93 Gb), and D genome (3.95 Gb). The largest number of markers in genome-wide association studies (GWAS) is determined in the B genome, and the smallest in the D genome [119,124], which is primarily explained by genetic diversity and the size of genomes.

**Figure 1:**
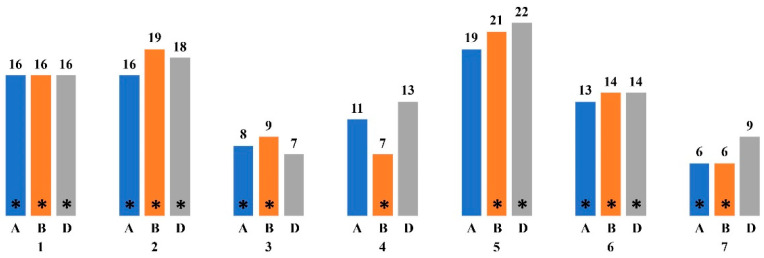
The distribution of the genes characterized as involved in photosynthesis along the chromosomes of *T. aestivum* based on the NCBI Genes database (https://www.ncbi.nlm.nih.gov/gene (accessed on 5 June 2024)). * indicates chromosomes on which loci associated with ChlF parameters and chlorophyll content have been identified; the letters A, B and D indicate genomes; 1–7 indicate chromosomes; the number of genes described is indicated by the numbers above the corresponding columns.

ChlF parameters reflect the activity of photosynthetic processes for which certain genes are responsible. The peculiarity of photosynthetic genes is their movement from the chloroplast genome to the nuclear genome during evolution, while maintaining functional activity directly in the chloroplasts. To date, it has been determined that about 10–15% of plant genes are involved in photosynthesis [105]. However, GWAS analyses of traits associated with photosynthetic activity often identify single nucleotide polymorphisms (SNPs) associated with more than just photosynthesis [125]. An analysis, performed on the basis of the NCBI Genes database (https://www.ncbi.nlm.nih.gov/gene (accessed on 5 June 2024)), revealed trends in the distribution of *T. aestivum* photosynthesis-related genes along chromosomes (Figure 1). The distribution of genes between genomes is even, but the largest number of genes is in the D genome and linkage groups 1, 2, 5 and 6.

Loci associated with fluorescence parameters and chlorophyll content were identified on chromosomes 1A, 1B, 1D, 2A, 2B, 2D, 3A, 3B, 4B, 5B, 5D, 6A, 6B, 6D, 7A, 7B. Particularly, chromosomes from the B genome are often described as including loci associated with photosynthetic parameters and chlorophyll in bread wheat and triticale [126,127,128,129,130,131]. The loci associated with chlorophyll content under stress conditions such as drought are found on chromosomes 1B, 2A, 2B, 3A, 3B, 4A, 5A, 6B, 6D and 7B [106,107]. Under the influence of heat stress, such loci are identified on chromosomes 1A 2B, 2D, 4B, 4D, 5D, 6A and 6B [128,131].

ChlF parameters, reflecting the activity of photosynthetic processes, are polygenic traits, like many important classical breeding traits such as yield and tolerance to stress. The quantitative trait loci (QTLs) identified in studies are associated not only with ChlF parameters, but also with other significant traits. Seven QTL clusters associated with both photosynthesis and GY, including those limited by the marker pairs *Xgwm335.2*–*Xgwm186* and *Xgwm296.3*–*Xbarc168*, have been identified [132]. QTLs for F_v_/F_m_ and grain weight per ear were identified on chromosome 6B [127]. The region of the stable SNP peak Excalibur_rep_c68899_1400 on chromosome 2B is described as being responsible for Φ_PSII_ and wheat yield [133]. The locus in the *Xbarc99*–*Xbarc169* region on chromosome 1D was responsible for the net rate of photosynthesis and GY, and the locus in the *Xbcd1095*–*Xfbb113* region on chromosome 2B was additionally responsible for chlorophyll content [129]. Under drought conditions, shared loci have also been identified for ChlF parameters and GY on chromosome 7D [133], as well as ChlF parameters and drought tolerance in wheat on chromosome 1B [134].

The heritability of ChlF parameters should be discussed in detail. The results available to date show both the strong and weak heritability of ChlF parameters. In particular, a strong heritability of ChlF parameters has been shown in field studies on *Hordeum vulgare* plants [112]. At the same time, a low heritability of ChlF parameters was found on *T. aestivum* plants [52]. However, in this case, a limited number of measurements during each growing season should be taken into account. Considering the variability in weather conditions (in different cultivation years), the high sensitivity of ChlF parameters to environmental conditions may be a possible reason for the conclusion of the low heritability of traits. The above-mentioned high heritability of ChlF parameters under controlled conditions [112] supports this assumption.

**Figure 2:**
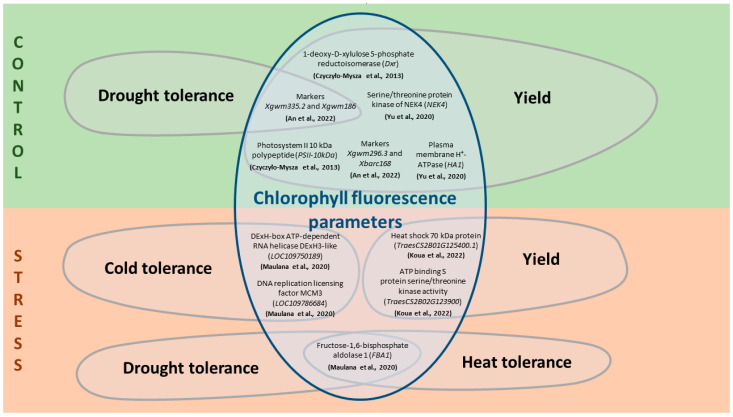
Relationship between ChlF parameters and significant breeding traits through some candidate genes under control and stress conditions for *T. aestivum*. The scheme is based on data from [127,129,132,133,135], a detailed description of the measured ChlF parameters and associated genes is described in Table S1.

To analyze the interaction of traits associated with ChlF and significant breeding traits, it is worth noting the candidate genes localized in regions of interest (Figure 2 and Table S1). The following gene ontology terms are described for most of the identified genes: ATP binding, protein phosphorylation, photosynthesis, electron transfer, oxidoreductase activity, and chaperone proteins [124,133]. The genes localized in the regions associated with the considered traits have been described both under optimal plant growth conditions and under stress conditions [127,128,133].

Under non-stress conditions, the SNP (IWB14950) for chlorophyll content is located in a region of chromosome 1B that has sequence similarity to the heat shock protein (*LOC109759017*), involved in heat stress tolerance [128]. On chromosome 1D, the SNP locus IWB17397 for GY and net photosynthetic rate contained the serine/threonine protein kinase NEK4 gene [129]. On chromosome 2B, the SNP locus IWA1040 for GY, net photosynthetic rate and, additionally, chlorophyll content, is associated with the plasma membrane H^+^-ATPase gene [129]. QTLs for F_v_/F_m_ were found on chromosomes 2A, 3A, 6A, 7A, 2B, 5B, 6B, 1D and 2D [127]. Moreover, three of the five regions on chromosome 6B contained QTL for ChlF parameters that also coincided with QTL for plant yield parameters [127]. The identified candidate genes are involved in photosynthesis and energy metabolism [127].

Under drought conditions, multiple significant SNPs associated with the DNA replication licensing factor MCM3 gene, which is involved in the formation of tolerance to salt and cold stress in other crop species, were identified for chlorophyll content on chromosome 1B [135,136]. Also, under the influence of drought, two significant SNPs (IWB60417 and IWB11846), associated with candidate genes (DExH-box ATP-dependent RNA helicase DExH3-like (*LOC109750189*) and the fructose-1,6-bisphosphate aldolase 1 (*FBA1*), respectively) were identified for F_v_/F_m_ on chromosome 3A [135]. The first gene is involved in plant development and tolerance to abiotic stresses, such as low temperature and freezing [137], while the latter is involved in plant stress reactions, such as drought and heat stress responses [138]. A locus for the combination of ChlF and yield traits has been identified on chromosome 2B. The locus, which has a pleiotropic effect for the quantum yield of PSII and GY under drought conditions, contained 31 genes responsible for the activity of peroxidase, nonspecific serine/threonine protein kinase and heat shock proteins [133]. In tetraploid wheat *T. timopheevii*, changes in photosynthesis parameters and yield under drought conditions caused by the activity of several genes localized in the distal region of the short arm of chromosome 2A [139]. *TraesCS2A01G044900.1*, encoding cytochrome P450, may be one of the candidates for plant stress response and yield effects [140]. *TraesCS2A01G035100.1* encodes the chloroplast protein CP12, which has been shown to be involved in the regulation of the activity of glyceraldehyde-3-phosphate dehydrogenase (GAPDH) and phosphoribulokinase (PRK) [141,142]. *TraesCS2A01G355400.1* encodes S-adenosylmethionine decarboxylase, an enzyme in the biosynthesis of polyamines involved in the regulation of tolerance to oxidative stress caused by drought [143]. Under heat stress, chlorophyll content markers are found on several chromosomes, including 2D, where candidate genes involved in stress responses and growth regulation are localized [128]. For example, plastid lipid-associated protein is involved in increasing plant yield under stress [144]. The N-terminal domain of heat shock protein is involved in plant responses to various environmental stresses, including heat [128]. IAA-amino acid hydrolase ILR1-like hydrolyzes amino acid conjugates of IAA (indole-3-acetic acid), which acts as a plant growth regulator [145]. Also, under heat stress, a gene encoding the K⁺ antiporter, which is involved in the response of plants to salt stress [146], was found on chromosome 4B [128].

It is worth noting a recently discovered pattern confirming the genetic relationship between ChlF parameters and significant breeding traits. An increase in the efficiency of photosynthesis among created cultivars occurs simultaneously with an increase in breeding progress. This is possible due to the selection of wheat cultivars that have allele variations that contribute not only to improved yield, but also to increased photosynthetic activity. For example, modern cultivars released after 2010 that have shown higher yields also have a higher chlorophyll content and quantum yield of PSII than cultivars released before 1980. Interestingly, for the quantum yield of PSII, the difference between these two groups was greater under drought than in control conditions, suggesting that selection has increased wheat yield under drought conditions through the accumulation of genetic variants that have a positive effect on photosynthetic activity under suboptimal conditions [133].

## 6. Conclusions

The method for recording ChlF parameters, based on PAM fluorometry, has high potential for use in breeding. One of the fields of its application is the assessment of the tolerance of genotypes to the effects of unfavorable factors during the growing season or when modeling stressors under controlled conditions. The prediction of economically significant traits at early stages of plant development is another potential niche for including ChlF parameters in the breeding process. In these cases, ChlF parameters can be used both as independent indicators of the potential of the genotypes under study and in combination with other phenotypic and genotypic characteristics. It is more appropriate to measure these parameters as predictors under controlled conditions due to the high influence of environmental factors on their values. Integrating PAM fluorometry into the process of selecting promising genotypes requires a deep understanding of the physiological basis of the relationship of ChlF parameters with the productivity potential of agricultural plants and with the nature of their response to changes in environmental conditions during the growth period, which directly affects the yield. In particular, a detailed study of the contribution of changes in the activity of a certain physiological process to the modulation of ChlF parameters and their impact on the final yield is necessary. It is also necessary to identify a set of genetic markers associated with parameters recorded using PAM fluorometry. This will provide a more meaningful and reliable selection of genome editing points or genotypes for traditional crossbreeding.

## Figures and Tables

**Table 1:** Commonly used ChlF parameters. Prepared by the authors based on data from Maxwell and Johnson [15], Baker [17]; Kalaji et al. [11,12]; and Klughammer and Schreiber [35].

Parameter		Calculation	Description
**Measured Parameters**			
$F_{0}$	Minimum fluorescence level in the dark-adapted state		Minimum fluorescence level excited by measuring light in the dark-adapted state (all PSII reaction centers are open)
$F_{m}$	Maximum fluorescence level in the dark-adapted state		Maximum fluorescence level excited by a saturation light pulse in the dark-adapted state (all PSII reaction centers are closed)
$F_{0}^{\prime }$	Minimum fluorescence level in the light-adapted state		Minimum fluorescence level excited by measuring light after illumination (when all PSII reaction centers are open, but non-photochemical quenching contributes to the decrease in fluorescence level)
$F_{m}^{\prime }$	Maximum fluorescence level in the light-adapted state		Maximum fluorescence level excited by a saturation light pulse after illumination (all PSII reaction centers are closed, but NPQ contributes to the decrease in fluorescence level)
$F_{t}$	Steady-state fluorescence level under illumination		Fluorescence level excited by actinic light (both photochemical and non-photochemical quenching contribute to the decrease in fluorescence level)
**Calculated parameters**			
$F_{v}/F_{m}$	Maximum quantum efficiency of PSII	$\frac{F_{v}}{F_{m}}=\frac{F_{m}-F_{0}}{F_{m}}$	Maximum efficiency of the photochemical use of the light absorbed by PSII
$\Phi_{\mathrm{PSII}}$ ($F_{q}^{\prime }/F_{m}^{\prime }$, YII)	Effective photochemical quantum yield of PSII	$\Phi_{\mathrm{PSII}}=\frac{F_{m}^{\prime }-F_{t}}{F_{m}^{\prime }}$	Estimates the proportion of the light absorbed by PSII that is used in photochemistry
qP ($F_{q}^{\prime }/F_{v}^{\prime }$)	Coefficient of photochemical fluorescence quenching	$\mathrm{qP}=\frac{F_{m}^{\prime }-F_{t}}{F_{m}^{\prime }-{\;F}_{0}^{\prime }}$	Estimates the proportion of open PSII reaction centers
ETR	Electron transport rate	$\mathrm{ETR}\;=\Phi_{\mathrm{PSII}}\;\times \;\mathrm{ETR}\;-\mathrm{Factor}\;\times \;\mathrm{PPFD}\;\times {\mathrm{fraction}}_{\mathrm{PSII}}$	Estimates the rate of the electron transport through PSII *ETR-Factor*—the fraction of incident photons absorbed by the leaf (usually taken equal to 0.84); *PPFD*—photosynthetic photon flux density; $fraction_{PSII}$—the fraction of PPFD absorbed by PSII (usually taken equal to 0.5).
NPQ	Non-photochemical fluorescence quenching	$\mathrm{NPQ}=\frac{F_{m}}{F_{m}^{\prime }}-1$	Estimates the rate constant for heat dissipation from PSII relative to the dark-adapted state
NPQ_F_	Fast-relaxing NPQ	${\mathrm{NPQ}}_{F}=\frac{F_{m}}{F_{m}^{\prime }}-\frac{F_{m}}{F_{m}^{r}}$	The energy-dependent component of NPQ
qN	Coefficient of non-photochemical fluorescence quenching	$\mathrm{qN}=1-\frac{F_{v}^{\prime }}{F_{v}}$	Estimates the rate constant for heat dissipation from PSII relative to the dark-adapted state; requires measurement of F_0_
Y(NO) $\left(\Phi_{\mathrm{NO}}\right)$	Quantum yield of non-regulated non-photochemical energy loss in PSII	$Y\left(\mathrm{NO}\right)=\frac{F_{t}}{F_{m}}$	Estimates the fraction of the light energy that is passively dissipated in form of heat and fluorescence

## Supplementary Materials

The following supporting information can be downloaded at: https://www.mdpi.com/article/10.3390/plants13192778/s1, Table S1: Relationship between ChlF parameters and significant breeding traits through some candidate genes under control and stress conditions for *T. aestivum*.
